# High-voltage electrical burn with median nerve salvage using Axoguard Nerve Protector and anterolateral thigh free flap: a case report

**DOI:** 10.1093/jscr/rjag614

**Published:** 2026-07-21

**Authors:** Sophia Kermet, Luke A Hudson, Brenda M Hranec, James D Kotick

**Affiliations:** Department of Clinical Sciences, Florida State University College of Medicine, 1115 West Call Street, Tallahassee, FL 32306-4300, United States; Department of Clinical Sciences, Florida State University College of Medicine, 1115 West Call Street, Tallahassee, FL 32306-4300, United States; Department of Clinical Sciences, Florida State University College of Medicine, 1115 West Call Street, Tallahassee, FL 32306-4300, United States; Envision Plastic and Reconstructive Surgery, 1561 Lakefront Dr Suite 202, Sarasota, FL 34240, United States

**Keywords:** electrical injuries, free tissue flap, median nerve injury, microsurgery, peripheral nerve regeneration

## Abstract

High-voltage electrical burns present as complex peripheral nerves damage, with complete recovery of nerve function rarely seen, especially in mixed motor and sensory innervation injury. Current treatments such as autografts carry the risk of donor site morbidity and potential neuropathy. In this case, a 35-year-old line worker sustained an electrical burn while at work and examination revealed a large volar burn along the right arm, hand, left hand, chest, and leg. After debridement, exposure of the radial artery, median nerve, and flexor tendons in the right upper extremity was noted. Further operative examination confirmed compartment syndrome of the volar compartment. The median nerve was identified as extensively damaged but physically intact. A multimodality approach was taken to avoid donor site morbidity, with a right upper extremity fasciocutaneous anterolateral thigh free tissue transfer and an Axoguard Nerve Protector (40 × 7 mm). Functional recovery was demonstrated at 8-month follow up.

## Introduction

Treatments for peripheral nerve damage include primary repair, autologous nerve grafts, nerve conduits, and tendon transfers. Surgical intervention options depend on the type of damage delivered to nerves, each presenting with unique biophysical challenges [1]. Distal median nerve repair is particularly complex due to long nerve gaps and high risk for neuroma-in-continuity [2–4].

Loss of median nerve function results in profound impairment of precision grip, sensation to the first three digits, and wrist flexion. Even after the median nerve is surgically repaired, patients frequently experience persistent motor deficits [2–4]. Functional limitations following repair result in social and economic consequences, particularly for working-age individuals whose livelihoods are dependent on manual dexterity.

A multimodality approach may offer a strategy with fewer associated complications [5]. Alternatives, such as Axoguard Nerve Protector, have the potential to be wrapped around injured nerves and help preserve the fascicular microenvironment and reduce perineural scarring, highlighting new strategies in managing complex cases [5].

This paper presents a case describing the use of an Axoguard Nerve Protector and anterolateral thigh (ALT) flap in the surgical repair of a high-voltage median nerve injury complicated by compartment syndrome. The technique shows promise in comparable injuries, as evidenced by functional recovery at 8 months postoperatively.

## Case report

A 35-year-old line worker presented with a 3500-volt electrical burn from a power line. Preoperative assessment showed a large volar burn along the right arm, hand, left hand, chest, and leg. Preoperative examination demonstrated active and preserved digital flexion and extension, supported by an intact flexor pollicis longus function, although examination was limited by volar compartment swelling. Sensory function was diminished throughout the entirety of the right hand. Following debridement, exposure of the radial artery, median nerve, and flexor tendons in the right upper extremity was noted. Further operative examination confirmed compartment syndrome of the volar compartment.

**Figure 1:**
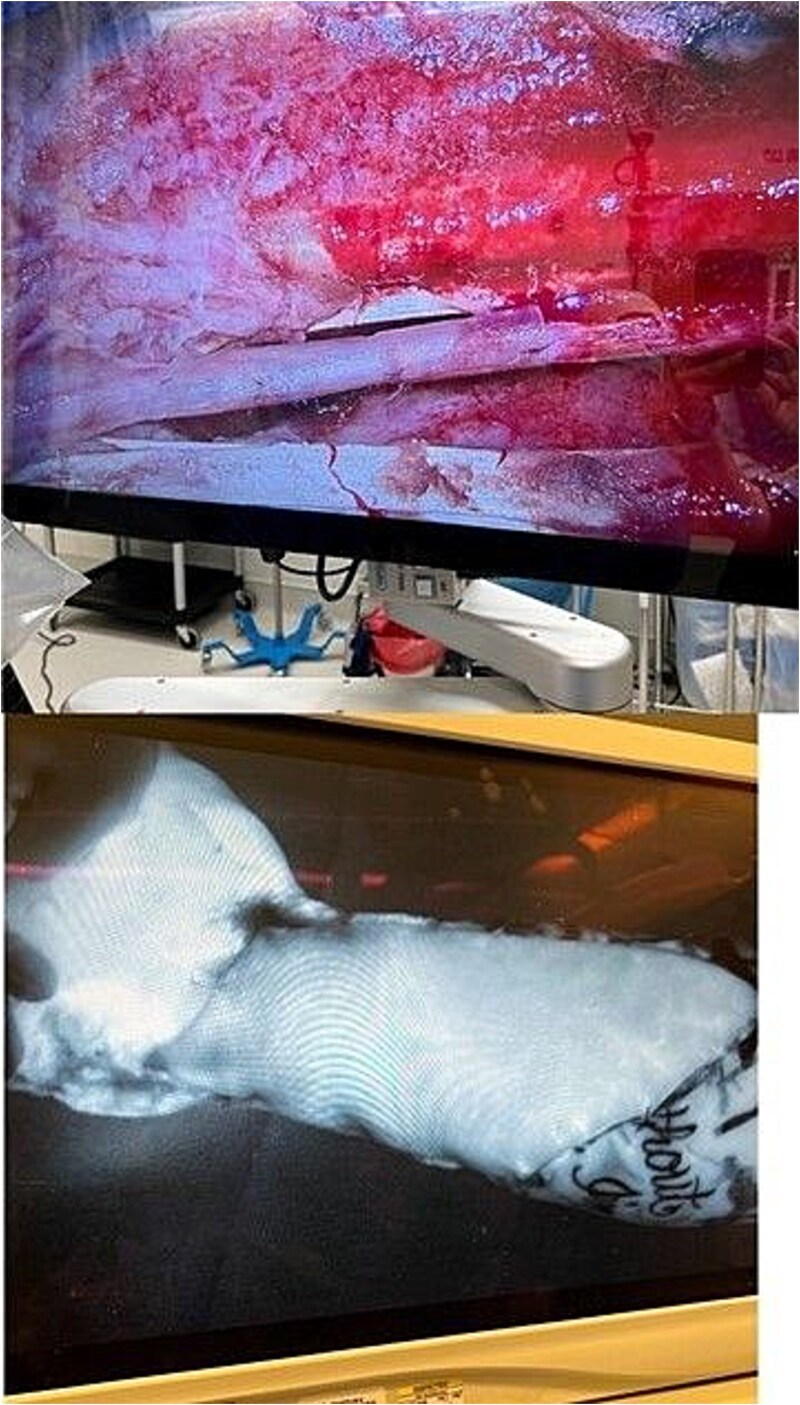
Salvaged median nerve with Axoguard Nerve Protector and covered by ALT free flap during surgical operation.

An emergent decompressive fasciotomy was performed on the right arm and forearm and the volar and dorsal compartments were released. Intraoperative exploration and excision of burned tissues revealed gross continuity of the median nerve without evidence of transaction within viable muscle and tissue (Fig. 1). Attention was turned to the thigh, and the dominant perforator was identified. indocyanine green (ICG) angiography was injected to validate that the skin flap had a strong and healthy blood flow supply. An ALT flap was harvested and prepared for coverage. An Axoguard Nerve Protector was wrapped around the median nerve to protect and promote axon regeneration (Fig. 1).

**Figure 2:**
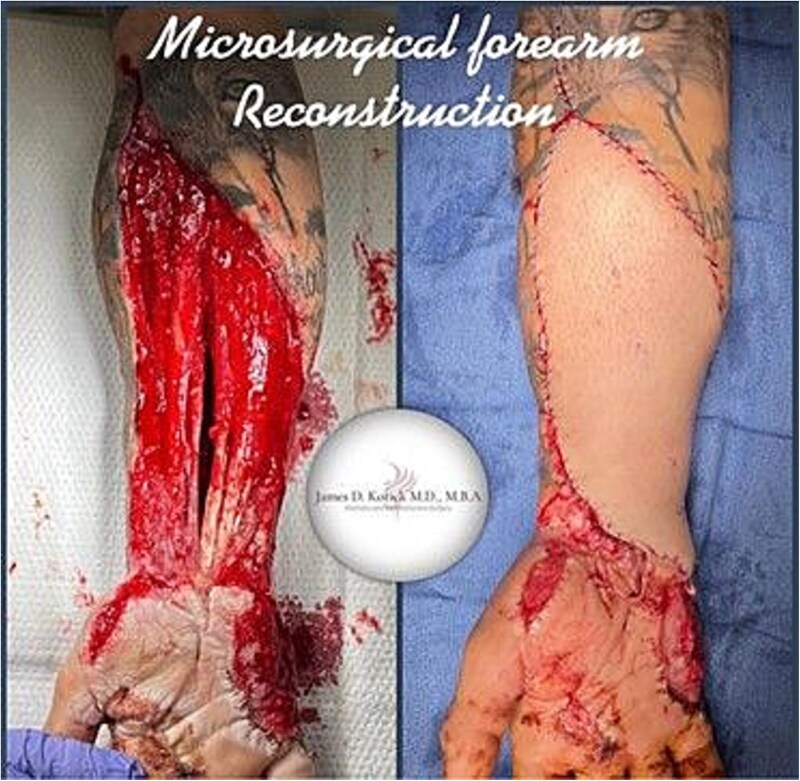
Intraoperative photograph demonstrates the right volar forearm before and after anterolateral thigh flap coverage was placed.

Preoperative Allen’s test and ultrasound confirmed ulnar dominance to the hand and thumb. The exposed radial artery was noted to be thrombosed in the wound bed. The radial artery was anastomosed to the descending branch of the lateral circumflex artery and the flap was inset (Fig. 2). ICG was injected and confirmed successful blood flow was achieved. Postoperatively, the patient was followed for nerve function and flap viability. Routine visits demonstrated slow, consistent improvement in sensation throughout the hand.

**Figure 3:**
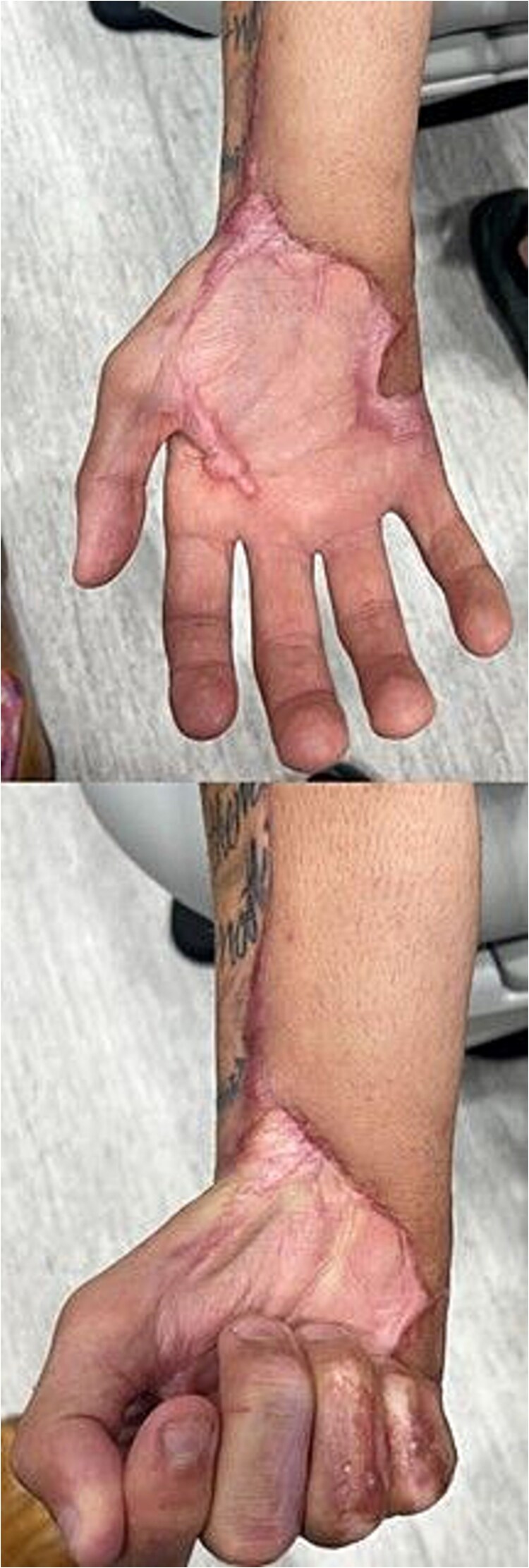
Eight-month follow up showing healed reconstruction of right volar forearm with preserved flap viability. No evidence of wound breakdown. Closed grip was achieved.

At 8-month follow up, with physical and occupational therapy, range of motion gradually improved and a closed grip was achieved (Fig. 3). The flap was well perfused and viable in follow-up sessions, with the donor site healed completely. Long-term follow-up is ongoing.

## Discussion

Current treatments for peripheral nerve damage include primary repair, autologous nerve grafts, nerve conduits, and tendon transfers. Challenges in median nerve repair have led to a rise in advances in microsurgery to improve functional outcomes [6]. The gold standard for peripheral injuries is primary nerve repair [1, 6]. Nerve grafts, such as autologous nerve grafts, are used to bridge physical nerve gaps, however, carry the potential for neuropathic pain and donor site morbidity. Nerve transfers are more commonly utilized for proximal injuries where axons must regenerate across long distances toward their targets and risk irreversible motor endplate degeneration. As such, nerve transfers are more applicable to injuries sustained at the level of the brachial plexus and are less ideal for distal median nerve injuries within the volar compartment [6].

This case highlights the advances made in nerve regeneration and salvage for distal peripheral nerve injuries, particularly in the setting of electrical burn trauma where reconstructive guidelines remain limited. Electrical injuries differ from sharp or crush mechanisms in that nerves may remain structurally intact yet biologically compromised due to ischemia, microvascular thrombosis, and progressive fibrosis. The environment in which the median nerve was identified was damaged but remained in continuity. To avoid harvesting an autologous nerve graft and exposing the patient to donor site morbidity, a synergistic multimodality reconstructive approach was utilized. An Axoguard Nerve Protector was used to protect the injured nerve and preserve the regenerative microenvironment, while an ALT free flap was utilized to provide vascularized soft tissue coverage. This novel combination played critical roles in mitigating ischemia, reducing fibrosis, and supporting the metabolic demands of regenerating nerves.

Intraoperative ICG angiography interpretation was employed at two distinct stages: first, to confirm perfusion of the harvested ALT flap prior to transfer, and second, following inset, to validate patency of the microvascular anastomosis. This real-time perfusion assessment optimized flap viability and ensured adequate vascular support to the underlying nerve.

Current evidence specific to electrical burn median nerve salvage remains scarce. Future investigation into integrated reconstructive paradigms may offer viable alternatives to autologous nerve grafting while expanding salvage options for complex distal peripheral nerve injuries.

In conclusion, advances in microsurgery show the promise of improving outcomes in severely damaged nerve cases, such as those following high-voltage electrical trauma. Surgeons are faced with diverse challenges in selecting appropriate clinical interventions depending on the location, severity, and type of injury present. In this case utilization of an ALT flap and Axoguard Nerve Protector acted synergistically to support axonal growth of the damaged but intact median nerve. This technique for high-voltage nerve repair demonstrates promise, with the repaired nerve showing functional capacity and continual improvement during the patient’s follow-up visit. Such advances in microsurgery allow measures to be taken to salvage native nerves and bypass the need to harvest donor nerves, thereby reducing donor site morbidity associated with traditional autografting.
